# GERIATRICS TRAUMA CO-MANAGEMENT: PRELIMINARY DESCRIPTIVE DATA FROM A SINGLE ACADEMIC INSTITUTION

**DOI:** 10.1093/geroni/igad104.2984

**Published:** 2023-12-21

**Authors:** Yuji Yamada, Ko Harada, Fred Ko, Emily Chai, Luis Suarez-Rodriguez, Raymond Wedderburn, Omar Amir

**Affiliations:** Icahn School of Medicine at Mount Sinai, New York City, New York, United States; Beth Israel Medical Center, New York, New York, United States; Icahn School of Medicine at Mount Sinai, New York City, New York, United States; Icahn School of Medicine at Mount Sinai, New York City, New York, United States; Icahn School of Medicine at Mount Sinai, New York City, New York, United States; Icahn School of Medicine at Mount Sinai, New York City, New York, United States; Icahn School of Medicine at Mount Sinai, New York City, New York, United States

## Abstract

**Background:**

Traumatic injuries cause higher mortality and complications in older patients than in younger individuals. Orthogeriatric co-management programs improve outcomes and quality of care and may be a model for trauma management. We theorized that geriatricians could improve trauma care in older adults with the early identification of geriatric syndromes.

**Methods:**

A retrospective chart review was conducted for patients aged > 65 years admitted to the trauma surgery service at a Level 2 trauma center between October 2022 and January 2023, who were co-managed by geriatricians as part of a pilot program.

**Results:**

67 patients were included in this study. The mean age was 81 (65-99), and 38% were male. The most common injuries were rib fractures (39%), subdural hematomas (32%), and spinal fractures (10%). Geriatric syndromes identified and managed by a geriatrician included frailty (47%), major or minor cognitive impairment (48%), delirium (19%), malnutrition (13%), and polypharmacy (65%), with a mean number of 6.7 home medications. As per Beers criteria, 63% of patients were taking inappropriate medications. Geriatrician-led interventions in advance care planning included identification and documentation of healthcare proxies (i.e., increased to 87% from 11% on admission) and goals of care conversations (54%).

**Conclusions:**

Given the high prevalence of geriatric syndromes and complex medical and social needs in older patients with trauma, geriatric co-management may enable timely diagnosis and treatment of conditions that cause poor outcomes. Further research is warranted to assess the impact of geriatric co-management models on the long-term clinical outcomes in older individuals after injury.

